# Personality Type and Chronic Pain: The Relationship between Personality Profile and Chronic Low Back Pain Using Eysenck’s Personality Inventory

**DOI:** 10.3390/neurosci3040049

**Published:** 2022-12-13

**Authors:** William J. Hanney, Abigail T. Wilson, Travis Smith, Chandler Shiley, Josh Howe, Morey J. Kolber

**Affiliations:** 1 School of Kinesiology and Physical Therapy, University of Central Florida, Orlando, FL 32816, USA; 2 Department of Physical Therapy, Nova Southeastern University, Fort Lauderdale, FL 33314, USA

**Keywords:** low back pain, personality, catastrophizing, fear avoidance, disability

## Abstract

Background: Personality type plays a key role in how individuals respond to a variety of stimuli; however, it is unclear if there is a significant influence on pain perception. While pain is associated with many conditions, chronic low back pain (cLBP) is one of the most prevalent and debilitating problems in modern society. Treating this condition can be a challenge and clinicians must understand all factors that can influence pain perception. Purpose: The present study investigated the relationship between personality type and pain experience in patients experiencing cLBP. Methods: One hundred twenty-four participants completed the Eysenck Personality Inventory (EPI), which identifies two major components of the human personality, neuroticism, and extraversion. Participants also completed the Oswestry Disability Index (ODI), the Tampa Scale for Kinesiophobia (TSK), the Numeric Pain Rating Scale (NPRS), and the Pain Catastrophizing Scale (PCS). The association between pain and personality was determined with a Spearman Rank Correlation Coefficient. A hierarchical cluster analysis with Ward’s clustering method examined for subgroups of individuals based on these variables. Results: The neuroticism score (EPI-N) was found to have a statistically significant relationship with all pain outcome measures. This suggests that people exhibiting a neurotic personality type are likely to have more fear of movement (*p* = 0.001), greater catastrophizing behavior (*p* < 0.001), higher self-reported levels of disability (*p* < 0.001), and higher overall reported levels of pain (*p* = 0.046) than those with other, more stable personality types. Three clusters were derived with varying levels of pain-related factors and personality. Conclusions: Personality type appears to have an influence on many of the attributes associated with cLBP and may be a useful determinate in both prognosis and interventions.

## 1. Introduction

In the United States, chronic pain is a problem that impacts millions of people [1]. It has been estimated that approximately 20% of Americans live with chronic pain [1]. While pain is associated with many conditions, low back pain (LBP) is among the most disabling medical conditions [2]. It has been reported that up to 13.1% of Americans between the ages of 20 and 69 report the presence of chronic low back pain (cLBP) at a given time (point prevalence) [2]. Furthermore, the economic cost in disability payments, medical care, and lost worker productivity due to LBP is over USD 2 billion annually [3].

Personality has been defined as a collection of individual differences in patterns of thinking, feeling, and behaving [4]. Evaluation of personality type has been an area of research in the social sciences for decades, with the Myers–Briggs Type Indicator first emerging during World War II to promote cohesion and teamwork in nursing units [5]. Personality tests are designed to quantify elements of the human psyche and construct a common language through which to discuss these elements [6]. However, it is unclear how personality influences pain perception and perceived disability.

A recent review discussed the many contextual factors that frame the pain experience in patients with cLBP [7]. Socioeconomic status, level of education, and job satisfaction, as well as co-occurrence of mental health disorders such as depression and anxiety, have all been shown to impact the pain experience, especially as it relates to the development of chronic pain [1,2,3,4,5,6,7,8,9]. These findings confirm existing research showing connections between cLBP and psychological conditions, such as anxiety and depression [8,9].

The Eysenck Personality Inventory (EPI) is one of the many personality tests that has been used in the scientific literature. Versions of the EPI have been used in clinical research applications, with mixed modes of use and varying degrees of success [10,11,12]. However, some of these studies had limitations. The Gilchrist study had a large sample size (n = 143) but investigated episodes of acute LBP [11]. This type of pain experience is more likely to involve true tissue damage and inflammation and may not be as susceptible to psychological influence as cLBP [11]. The study by Lin et al. [10] found significant relationships between neurotic personality type and the presence of chronic pain, but it examined patients with cervical pain, not LBP. Furthermore, the study by Garcia-Torres et al. [12] investigated breast cancer survivors and, therefore, its results are not likely to generalizable to patients with musculoskeletal conditions.

The EPI identifies two major components of human personality, neuroticism and extraversion, and places them on a Cartesian coordinate system to classify personality type [13]. The interplay between these two components, neurotic vs. stable and introverted vs. extraverted, combine to classify one’s personality as Choleric (extraverted and neurotic), Sanguine (extraverted and stable), Phlegmatic (introverted and stable), or Melancholic (introverted and neurotic).

To the authors’ knowledge, there is a paucity of research investigating the association between personality type and pain experience in patients with LBP. The pain experience can be challenging to evaluate and can influence many different constructs. For example, individuals suffering from LBP may have increased difficulty performing normal daily activities and exhibit a degree of perceived disability. In addition, those in pain may demonstrate a certain degree of concern or fear with regard to movement. Therefore, the present study utilized the EPI as a tool to investigate the relationship between personality and perceived pain, disability, and fear of movement in those with cLBP. The primary purpose of this study was to determine the association between personality measured with the EPI and pain-related characteristics in individuals with cLBP. The secondary purpose of this study was to determine homogenous subgroups of this population based on personality, clinical factors, and pain-related psychological factors.

## 2. Materials and Methods

The present study was a prospective cohort survey. The survey instrument collected the following demographic data: age, height, weight, race, and sex. The survey also included the following validated outcome measures for measuring pain and disability: the Oswestry Disability Index (ODI), the Tampa Scale for Kinesiophobia (TSK), the 11-point Numeric Pain Rating Scale (NPRS), and the Pain Catastrophizing Scale (PCS), as well as the full EPI questionnaire [14,15,16,17,18].

**Figure 1:**
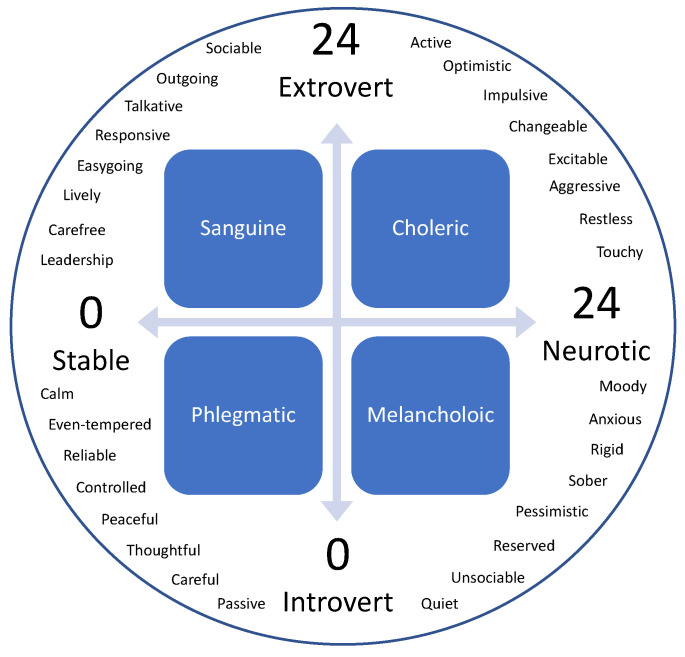
The Eysenck Personality Inventory (EPI). Note: The four quadrants in the figure above correspond to the four personality types outlined by the Eysenck Personality Inventory (EPI). The personality traits circling the figure are traits that are associated with each type.

The ODI includes 10 sections that highlight constructs or activities that those with low back pain may note as challenges. Each section is scored on a 0–5 scale, with 5 representing the greatest amount of perceived disability. The TSK is a 17-item scale intended to measure fear of movement related to lower back pain. The 11-point NPRS is a subjective measure in which individuals rate their pain on an eleven-point numerical scale ranging from 0 (no pain at all) to 10 (worst imaginable pain). NPRS data were collected for current, least, and worst pain intensity and then averaged to determine the NPRS for each participant. The PCS is a 13-item scale to assess catastrophic thoughts associated with low back pain according to 3 components: rumination, magnification, and helplessness; higher scores are associated with higher amounts of pain catastrophizing. The EPI is comprised of 57 yes or no questions: 24 questions for neuroticism, 24 questions for extraversion, and 9 questions designed to determine if the person taking the test is answering truthfully or not. A score of 0–12 on the extroversion scale would classify someone as introverted, whereas a score of 12–24 on the same scale would classify someone as extroverted. Similarly, a score of 0–12 on the neuroticism scale would classify someone as stable, whereas a score of 12–24 would classify someone as neurotic. As demonstrated in Figure 1, those scores can be plotted on the coordinate system, with the neuroticism scale corresponding to an *x*-axis, while the extroversion scale is a *y*-axis.

The test–retest reliability of the EPI ranges from 0.81 to 0.97 [13]. Another study found the internal consistency of each test section to be 0.89, 0.92, and 0.78 for the extraversion, neuroticism, and lie scores, respectively [18]. The pain, disability, fear of movement and catastrophizing outcome measures (ODI, TSK, NPRS, and PCS) have been used extensively in the literature and have been validated with patients experiencing LBP [14,15,16,17]. The survey was compiled into a comprehensive document and administered via Qualtrics software (Qualtrics, Provo, UT, USA). The study was approved by the University of Central Florida Institutional Review Board, and informed consent was obtained from participants.

The inclusion criteria for this study included adults over the age of 18 and currently experiencing chronic LBP. Individuals were excluded if they had a history of spine surgery or reported being pregnant. An a priori power analysis with an effect size of 0.25, alpha of 0.05, and power of 0.80 indicated that 128 participants would be required for this study to achieve appropriate statistical power. Participants were recruited using word-of-mouth and grass roots social-media marketing. Data collection began on 1 December 2021 and continued through to 4 December 2021. In total, 158 responses were collected. All data were de-identified to maintain participant confidentiality.

Data analysis for the primary purpose was completed using JASP open-source software, version 0.14.1 (University of Amsterdam). The primary purpose of this study was to determine the association between pain and personality in individuals with cLBP. Descriptive statistics were obtained for continuous data and frequencies for categorical data. The ODI, PCS, and TSK did not meet parametric assumptions, due to a lack of a normal distribution. Therefore, a Spearman’s rank correlation determined the direction and magnitude of the associations between the EPI and ODI, PCS, and TSK. Due to the ordinal nature of the data, a Mann–Whitney U test determined differences in the PCS and TSK by neurotic and stable personality types.

The secondary purpose of this study was to determine homogenous subgroups of individuals based on personality, clinical factors, and pain-related psychological factors. SPSS v. 28 (Armonk, NY, USA) was used for a cluster analysis to form subgroups, or “clusters”, that share similar characteristics. Forming homogenous subgroups within a heterogenous sample of individuals is a method of identifying inter-individual variability that may represent a first step toward greater precision in care. Clusters were formed based on personality (extroversion and neuroticism), clinical factors (pain intensity and disability), and pain-related psychological factors (catastrophizing and kinesiophobia). Sample size requirements vary for cluster analyses; however, recommendations suggest aiming for at least 20–30 participants per subgroup [19]. In preparation for the analysis, variables were first Z-transformed to standardize the metric across scales. A higher Z-score indicated a higher score on each measure. Current, best, and worst pain intensity were averaged to determine the average NPRS for each participant.

The following Z-transformed variables were entered into a hierarchical cluster analysis with Ward’s clustering method and squared Euclidean distances: extroversion scores on the EPI, neuroticism scores on the EPI, average NPRS, percent disability on the ODI, total PCS scores, and total TSK scores. These factors were selected to include measures of personality, pain-related psychological factors, and clinical factors. A hierarchical cluster analysis with Ward’s clustering method was selected, as the number of a priori clusters was unknown. Cluster determination was based on the largest change in agglomeration coefficients between two adjacent steps. As visual confirmation, the scree plot and dendrogram were inspected to confirm the number of clusters. Finally, cluster assignment was saved as a new variable in the dataset for each participant to allow for follow-up analyses further characterizing each cluster. A one-way ANOVA with cluster assignment as the between-subject factor was conducted to determine if clusters significantly differed by entered variables. An alpha level of 0.05 was considered statistically significant.

To characterize the derived clusters, differences across the subgroups were examined by demographic factors. A one-way ANOVA or chi-square analysis with cluster assignment as the between-subject factor determined if clusters significantly differed by continuous or categorical variables, respectively.

## 3. Results

One hundred twenty-four participants (65 male and 59 female) were included in the analysis. Thirty-four participants were removed from the analysis via listwise deletion, due to incomplete responses on surveys. Table 1 provides the descriptive data of participants.

**Figure 2:**
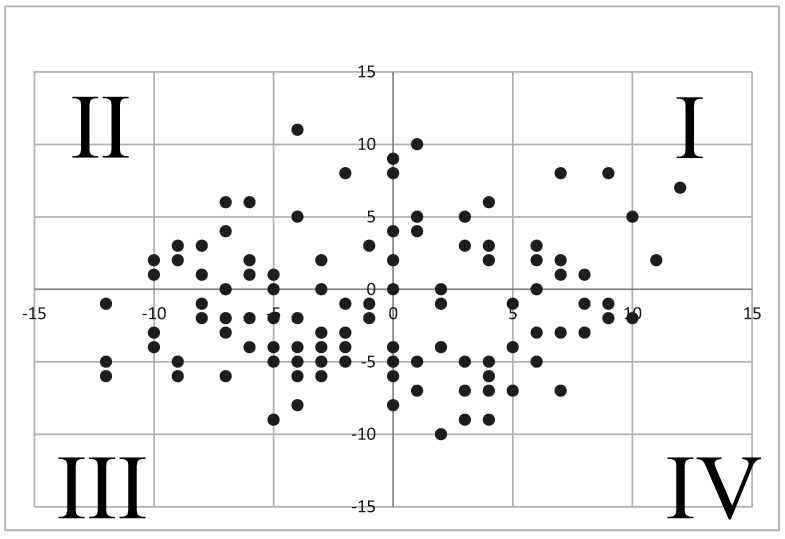
EPI profile. Note: This coordinate system represents the cross section of participants’ EPI-N and EPI-E scores. Each quadrant above corresponds to one of the four profiles identified by the EPI (refer to Figure 1): quadrant I is high neurotic/high extrovert (Choleric), quadrant II is low neurotic/high extrovert (Sanguine), quadrant III is low neurotic/low extravert (Phlegmatic), and quadrant IV is high neurotic/low extrovert (Melancholic).

Correlational analysis, using Spearman’s rank coefficients (ρ), revealed statistically significant relationships between the EPI-N score and the following pain-inclusive outcome measures: PCS score (*p* < 0.001, ρ = 0.381), ODI score (*p* = 0.038 ρ = 0.187), and TSK score (*p* = 0.008 ρ = 0.239). Collectively, neuroticism had a significant positive association with catastrophizing, disability, and kinesiophobia. A Mann–Whitney U test yielded significant differences between neurotic and stable personality types on the PCS (*p* < 0.001) and TSK (*p* = 0.012). Figure 2 and Table 2 present correlational results. Table 3 presents independent samples test results.

**Figure 3:**
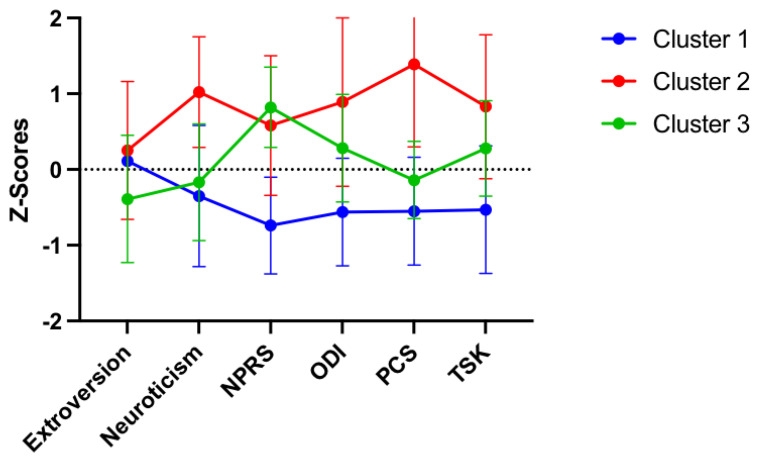
Differences across clusters by personality, clinical factors, and pain-related psychological factors.

These associations were supported by the results of the cluster analysis. Ward’s hierarchical cluster analysis resulted in three clusters (71% change in agglomeration coefficient between adjacent steps). The resulting clusters exceeded our sample size recommendations of 20–30 participants per group. Cluster 1 (n = 61) was the largest. As demonstrated in Figure 3, this subgroup included individuals with the least amount of neuroticism, low back pain intensity, and disability, as well as the lowest negative pain-related psychological factors. Cluster 2 (n = 27) was the smallest subgroup and consisted of individuals with high levels of neuroticism and high pain catastrophizing. This group had a lower pain intensity, compared with disability. Cluster 3 (n = 36) consisted of individuals with low extroversion and low pain catastrophizing. This cluster reported a higher pain intensity, compared with disability. Clusters significantly differed by these variables (*p*’s < 0.05). As demonstrated in Table 4, clusters did not significantly differ by age (*p* = 0.11), sex (*p* = 0.12), or race (*p* = 0.34).

## 4. Discussion

The positive correlation between EPI-N scores and PCS, ODI, and TSK scores suggest that those with a more neurotic personality type are also more likely to exhibit catastrophizing behavior, report higher levels of disability, and have higher levels of movement-related fear in those with LBP. The results of the independent samples t-test indicated similar tendencies: participants in this study classified as neurotic had higher average scores on both the TSK and PCS. This is consistent with Lin et al.’s [10] findings that neuroticism is positively correlated with the presence of chronic neck pain. Lin commented further on the association of general psychiatric distress and anxiety being associated with the presence of chronic neck pain [10,16].

All correlations between EPI-N scores and pain outcome measures, even those that lacked statistical significance, were positive. This aligns with Garcia-Torres et al.’s [12] conclusion that suffering through the ordeal of cancer diagnosis and treatment may have a predictive influence on neurotic personality type. This hypothesis suggested that the experience of going through cancer diagnosis and treatment may have preceded, or even led to the development of, a neurotic personality type [10]. The results of the present study did not measure change over time, so it is unclear whether participants were classified as neurotic before or after developing LBP symptoms.

There were no statistically significant relationships between EPI-E scores and any of the pain-related outcome measures. Despite none of these relationships amounting to statistical significance, Spearman Rho rank correlation coefficients classified them all as negative relationships. These results do not align with Gilchrist et al.’s [11] findings, which showed positive relationships between EPI-E scores and episodes of acute LBP. Gilchrist et al. [11] hypothesized that perhaps extraverted individuals were more likely to be active and, thus, more likely to suffer from spinal injuries as a result of relatively higher levels of physical activity, while prolonged episodes of LBP may be more likely to be associated with neurotic tendencies [12]. The results of the present study support this claim.

An additional finding of this study is the emergence of three pain and personality profiles or “clusters”. Each cluster represents significantly different levels of personality, pain intensity, disability, catastrophizing, and kinesiophobia. The first cluster was the largest and was characterized by a stable personality and decreased back pain intensity, disability, and negative pain-related psychological factors. The second cluster was characterized by a more neurotic personality and high levels of pain catastrophizing. The third cluster was characterized by high pain intensity but lower catastrophizing and kinesiophobia. Prior studies have determined homogenous subgroups of patients with LBP by psychological factors, pain sensitivity measures, and a combination of these variables [20,21,22,23]. This study adds to the body of literature by demonstrating that profiles differ by personality as well. Similar findings have been demonstrated in a sample of healthy individuals with experimentally induced pain [24,25]. Higher levels of neuroticism measured with the EPI were demonstrated in a cluster of individuals with high negative emotions. However, neuroticism decreased in a cluster of individuals with only high fear of pain [26]. Furthermore, the pattern of clusters suggest that varying personality levels and pain-related psychological factors exist among patients with cLBP. Although personality remains stable over time, it may be an additional factor to consider in examining the inter-individual variability of pain. Future studies may aim to determine whether short- and long-term clinical outcomes differ by cluster. Homogenous subgroups of patients are clinically relevant as subgroups of patients with musculoskeletal pain, displaying different trajectories of outcomes [24,27,28,29,30]. While the cluster with the stable personality displayed lower pain and disability during this cross-sectional study, it is unknown if this group would display a greater magnitude of change in clinical outcomes in response to treatment. Collectively, three pain and personality profiles emerged in this study, supporting the role of personality in the pain experience for individuals with cLBP.

The current study establishes a relationship between personality type and various behaviors and perceptions surrounding pain experience in patients [30]. Clinically, this information can impact the style of care given to patients in pain. Even without administering an EPI to a patient, clinicians can observe whether a patient is moody, aggressive, anxious, or pessimistic. According to the EPI manual, these traits are associated with the neurotic personality profile, and these patients will be more likely to exhibit higher levels of catastrophizing and kinesiophobia, as well as to report higher levels of pain and disability.

Other studies have evaluated the influence personality may have on low back pain. One study evaluated personality characteristics for those with non-specific chronic low back pain and found positive correlations between central sensitization inventory scores and sensory hypersensitivity profiles and trait anxiety. This is consistent with our findings, which suggest that psychosocial roles may influence pain perception [31]. In addition, it seems there is value in assessing personality traits to identify risk factors for psychological distress in challenging patients with low back pain [32]. This is one reason the authors selected the EPI, as it is relatively short and easier to incorporate into a clinical practice that treats individuals with low back pain. Additionally, some authors have utilized personality traits to identify individuals with a predisposition for developing chronic pain and this may provide a useful predictive screening instrument that may be an avenue for future investigations [33].

Limitations of the present study included our inability to reach the target sample size, missing the mark by four surveys. Another limitation was that recruitment for this study took place exclusively on social media, primarily within the social circles of the research team. Due to this circumstance, participants needed internet access and an appropriate device (computer or smart phone) to take part in the study. Furthermore, the survey was only available in English, and an overwhelming majority of respondents were white (76.6%), which prevented generalization to a broader society. Future studies could offer surveys in other languages and use different recruitment methods to obtain a more diverse, representative sample.

Areas for future research may include examining the individual personality types delineated by the EPI: Choleric, Sanguine, Phlegmatic, and Melancholic. The results of the present study show strong links between high neuroticism and more negative responses to pain. It would be of interest to carry out analyses on individual profiles characterized by the EPI.

Further areas of future research could examine different types of LBP. Idiopathic non-specific cLBP, in particular, is a pain experience that may be more susceptible than others to the influence by psychological factors, such as personality type. Similarly, it would be of interest to compare different types of treatments utilized for LBP based on personality profile to determine if a particular treatment approach may be better suited for a given personality.

## Figures and Tables

**Table 1:** Descriptive characteristics of the sample (N = 124).

	Mean (SD)	Min	Max
Age (years)	43.0 (18.2)	19	88
Height (inches)	68.4 (5.4)	60	105
Weight (pounds)	185.0 (47.9)	106	350
**Sex**	**N**	**Percent (%)**	
Male	65	52.4	
Female	59	47.6	
**Race**	**N**	**Percent (%)**	
Not specified	3	2.4	
White	95	76.6	
Black/African American	5	4.0	
Latino	10	8.1	
Asian	8	6.5	
Pacific Islander	3	2.4	

**Table 2:** Results of the Spearman’s rank correlation analysis between personality, clinical factors, and pain-related psychological factors.

	EPI-E	EPI-N
PCS	ρ = −0.026	ρ = 0.381
	*p* = 0.770	*p* < 0.001
ODI	ρ = −0.121	ρ = 0.187
	*p* = 0.182	*p* = 0.038
TSK	ρ = −0.081	ρ = 0.239
	*p* = 0.373	*p* = 0.008
NPRS-W	ρ = −0.068	ρ = 0.109
	*p* = 0.453	*p* = 0.230
NPRS-B	ρ = −0.082	ρ = 0.127
	*p* = 0.368	*p* = 0.161

Note: PCS: Pain Catastrophizing Scale, ODI: Oswestry Disability Index, TSK: Tampa Scale for Kinesiophobia, NPRS-W: Numeric Pain Rating Scale at the Worst, NPRS-B: Numeric Pain Rating Scale at the Best, EPI-E: Eysenck Personality Index Extraversion Score, EPI-N: Eysenck Personality Inventory Neuroticism Score.

**Table 3:** Independent samples test for neurotic vs. stable sttributes.

Variable	Mean Scores (SD)	*p*-Value	Effect Size
PCS	Neurotic: 11.483 (11.271)	*p* < 0.001	0.367
	Stable: 4.485 (5.339)		
TSK	Neurotic: 36.638 (8.733)	*p* = 0.012	0.261
	Stable: 33.288 (7.088)		

PCS: Pain Catastrophizing Scale, TSK: Tampa Scale for Kinesiophobia.

**Table 4:** Differences across clusters by demographic variables.

	Total Sample (n = 124)	Cluster 1 (n = 61)	Cluster 2 (n = 27)	Cluster 3 (n = 36)	*p*-Value	Effect Size
Age Mean (SD)	43.3 (18.2)	41.2 (18.2)	40.9 (16.6)	48.7 ± (18.5)	0.110	0.036
Sex (% female)	47.6	40.7	28.8	50.0	0.120	0.186
Race (%)					0.340	0.298
Not-specified	2.4	1.6	7.4	0		
White	76.6	75.4	63.0	88.9		
African-American	4.0	6.6	3.7	0		
Latino	8.1	6.6	11.1	8.3		
Asian	6.5	6.6	11.1	2.8		
Native Hawaiian or Other Pacific Islander	2.4	3.3	3.7	0		

## Data Availability

Not applicable.
